# Integration of Three-Dimensional Liver Models in a Multimodal Image-Guided Robotic Liver Surgery Cockpit

**DOI:** 10.3390/life12050667

**Published:** 2022-04-30

**Authors:** Okker D. Bijlstra, Alexander Broersen, Timo T. M. Oosterveer, Robin A. Faber, Friso B. Achterberg, Rob Hurks, Mark C. Burgmans, Jouke Dijkstra, J. Sven D. Mieog, Alexander L. Vahrmeijer, Rutger-Jan Swijnenburg

**Affiliations:** 1 Department of Surgery, Leiden University Medical Center, 2333 ZA Leiden, The Netherlands; r.a.faber@lumc.nl (R.A.F.); f.b.achterberg@lumc.nl (F.B.A.); j.s.d.mieog@lumc.nl (J.S.D.M.); a.l.vahrmeijer@lumc.nl (A.L.V.); 2 Department of Surgery, Amsterdam University Medical Center, Cancer Center Amsterdam, University of Amsterdam, 1081 HV Amsterdam, The Netherlands; r.j.swijnenburg@amsterdamumc.nl; 3 Section of Image Processing, Department of Radiology, Leiden University Medical Center, 2333 ZA Leiden, The Netherlands; a.broersen@lumc.nl (A.B.); j.dijkstra@lumc.nl (J.D.); 4 Section of Interventional Radiology, Department of Radiology, Leiden University Medical Center, 2333 ZA Leiden, The Netherlands; t.t.m.oosterveer@lumc.nl (T.T.M.O.); m.c.burgmans@lumc.nl (M.C.B.); 5 Department of Radiology, Amsterdam University Medical Center, 1081 HV Amsterdam, The Netherlands; r.hurks@amsterdamumc.nl

**Keywords:** robotic surgery, liver surgery, multimodal imaging, image-guided surgery, virtual reality, 3D

## Abstract

**Background:** Robotic liver surgery represents the most recent evolution in the field of minimally-invasive liver surgery. For planning and guidance of liver resections, surgeons currently rely on preoperative 2-dimensional (2D) CT and/or MR imaging and intraoperative ultrasonography. Translating 2D images into digital 3-dimensional (3D) models may improve both preoperative planning and surgical guidance. The da Vinci^®^ robotic surgical system is a platform suitable for the integration of multiple imaging modalities into one single view. In this study, we describe multimodal imaging options and introduce the Robotic Liver Surgery Cockpit; **Methods:** in-house developed software was used and validated for segmentation and registration to create a virtual reality 3D model of the liver based on preoperative imaging. The accuracy of the 3D models in the clinical setting was objectively assessed in 15 patients by measuring tumor diameters and subjectively with a postoperative conducted questionnaire; **Results:** Implementation and applicability of the 3D model in the surgical cockpit was feasible in all patients and the quality of the 3D reconstructions was high in 14 (93%) of cases. Tumor diameters measured on CT and/or MR imaging were comparable to automated measurements using the segmentation software and 3D models; **Conclusions:** the 3D model was successfully incorporated in the robotic surgery console as part of a multimodality imaging platform and aided the surgeon in planning and guidance of the resection. Future studies should focus on further automation of 3D rendering and progress into augmented reality.

## 1. Introduction

In recent years, hepatobiliary surgery moved from open procedures to an increasing number of minimally invasive approaches with robotic liver surgery as the most recent evolution [1,2]. Benefits of the robotic approach for liver surgery include reduced intraoperative blood loss, fewer complications, lower conversion rates, and shorter length of hospital stay [3,4,5]. In addition, the robotic surgical console can serve as a platform for imaging integration. Since tactile and, sometimes, haptic feedback is lacking in minimally invasive resections, surgeons increasingly rely on additional preoperative and intraoperative tools for surgical planning and surgical guidance prior to and during liver surgery. Preoperative contrast-enhanced computed tomography (CT) and/or magnetic resonance (MR) imaging are indispensable tools in diagnosing liver lesions [6]. However, these 2-dimensional (2D) preoperative diagnostic images acquired from CT and/or MR scans have to be mentally translated by the surgeon into 3-dimensional (3D) to match 2D scans with real 3D anatomy for adequate planning and guidance of surgical procedures. The latter may be challenging because multiple vascular and biliary anatomical variations exist in hepatic anatomy [7,8,9]. The success of surgical procedures is highly dependent on the surgeon’s ability to mentally create a 3D reconstruction of the liver.

Segmentation of preoperative CT and/or MR imaging scans and subsequent generated virtual reality 3D models may provide a clear visualization of the liver parenchyma, tumors, vital anatomical structures, and the relation of the tumor(s) to these vital structures. The applicability of 3D models was already described as early as 2010 in open liver surgery for preoperative planning of liver resection and living donor transplantation [10,11,12,13].

The role of virtual reality and augmented reality 3D models during robotic liver surgery has only been described in small case studies [14,15,16]. Robot-assisted procedures are especially suitable for the integration of multimodal imaging tools, since the surgical console offers the option of running multiple software applications in parallel, with a simultaneous view of the surgical field. The use of 3D reconstructions for the planning of robot-assisted partial nephrectomy has been extensively studied in large cohort studies and randomized trials with proven added value [17,18]. Specifically, for robot-assisted liver surgery, data on the added value of virtual reality 3D, and integration with other intraoperative imaging tools (e.g., ultrasound, fluorescence imaging) and models are lacking.

In order to aid in preoperative planning, the line-up, and adequate port placement of the robotic trocars and intraoperative surgical guidance, an in-house developed software program was designed to create virtual reality 3D models of the liver based on preoperative CT and/or MR imaging.

Here, we describe the creation and validation of in-house developed 3D modeling of the liver, and we introduce the Robotic Liver Surgery Cockpit, a multimodality imaging platform using intraoperative 3D liver modeling, ultrasound, and ICG-fluorescence imaging, integrated into the robotic surgical console.

## 2. Materials and Methods

### 2.1. Development and Validation of the 3DeliverS Software

Anatomical structures from different imaging modalities were segmented, co-registered, and combined into a patient-specific 3D model using an in-house developed software application that was created in the MeVisLab framework (version 3.4.2 MeVis Medical Solutions AG, Bremen, Germany). Each structure was segmented differently with an automatic, semi-automatic or manual method. A co-registration method was applied to anatomically align scans from different time-points or different modalities.

The liver was automatically segmented from CT scans by a deep learning approach using a 2D U-net that was trained using the data from the LiTS Challenge. The trained network can be used for liver delineation on a system with or without a GPU (CUDA 9.2) using TensorFlow (version 1.10). All or a subset of axial slices were offered to the network, after which the segmentation results were transformed into a contour. Any segmentation mistakes in the contours could be easily manually corrected. If MR imaging was used for liver segmentation, a number of contours were manually drawn and these contours were subsequently automatically interpolated. All tumors were manually segmented by an expert on every axial slice to ensure accuracy. The arterial, hepatic venous, and portal venous vasculatures were semi-automatically segmented using a region-growing approach with one or multiple seed points that were placed in the base of the vascular tree on the arterial, venous, and portal phase, respectively.

#### 2.1.1. Image Registration Methods and 3D Modeling

An automatic 3D co-registration method (Elastix version 5.0, GitHub, San Francisco, CA, USA) was integrated in the application to be able to combine the segmented structures from different scans or modalities. The segmented liver was used as a mask to indicate the region of interest on which the advanced Mattes mutual information similarity measure was applied to optimize the rigid registration approach. Optionally, manual translations and rotations could be applied prior to the automatic refinement of this initial co-registration.

The 2D contours with the segmentation results were converted into a volumetric mask. With these 3D masks, surfaces of the segmented structures (liver, tumors, gallbladder, arterial vasculature, portal and hepatic veins, vena cava and bones) were extracted using a marching cubes approach. Triangles were generated for each voxel that belongs to one structure, after which, these triangles were combined into one 3D surface. These 3D models were visualized using ParaView (version 5.9.0, Kitware, New York, NY, USA) or the web version ParaView Glance (version 4.18.2, Kitware, New York, NY, USA) by converting the 3D scene into the vtkjs-format for visualization on a tablet or smartphone.

#### 2.1.2. Validation

The software was validated using the commercially available NEMA-2012 PET phantom (NEMA-2012). The NEMA-2012 phantom contains six spheres with a known diameter, making it suitable for validation of segmentations in the 3DeliverS software. CT scans of the phantom were already available and were acquired with the Philips Vereos Digital PET/CT, Philips Eindhoven, the Netherlands. The in-plane resolution of the scan was 1.17 mm × 1.17 mm with a slice thickness of 0.67 mm. All spheres were segmented by two independent observers (AB and TO). Segmentation was performed by manually delineating at least the smallest and largest axial contours, using a B-spline contour algorithm. Interpolation was used to calculate all in-between contours. Subsequently, the interpolated contours were manually checked to be located on the inside edges of the spheres. Contours which were not located on the inside edges were manually corrected by the observer, followed by recalculation of the interpolation. This process was repeated until the observer was satisfied with the segmentation result. After segmentation, the contours were converted into a 3D model. The largest axial diameter and 3D volume were then extracted from 3DeliverS, and compared with the specifications according to the manufacturer.

### 2.2. Implementation of 3D Rendered Models

#### 2.2.1. Patients and Imaging

After completion of the validation tests, the software was used in a feasibility study in 15 patients planned for a robot-assisted liver resection. The protocol for this study was approved by the local institutional review board at Amsterdam UMC. No formal written informed consent was required according to the institutional review board. Intraoperative parameters, such as blood loss, operation time, and resection margin status from all included patients were documented.

**Figure 1:**
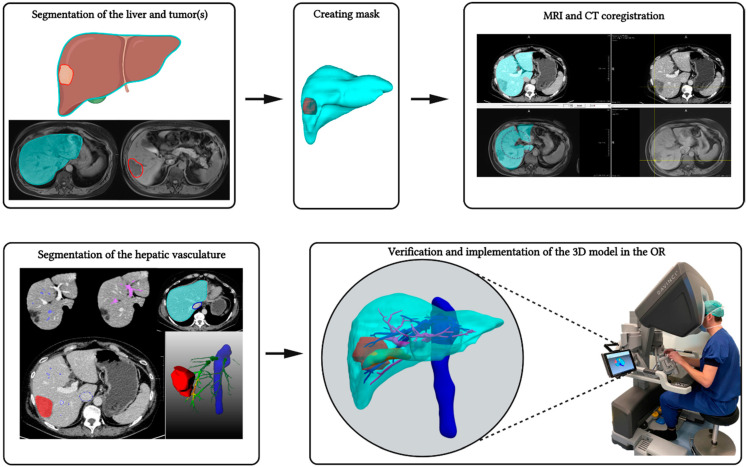
**Schematic overview of the segmentation process.** First, the liver and tumor(s) were segmented; subsequently, a mask was created. When the segmentation of the liver and tumor(s) was performed on MR imaging, MRI and CT co-registration was performed prior to the segmentation of the hepatic veins, portal veins, and cava vein. Finally, after all structures were segmented, a virtual reality 3D model was created. The relevant structures were verified by the surgeon, after which, the virtual reality 3D model was implemented in the Robotic Liver Surgery Cockpit. Created with BioRender.com.

Contrast-enhanced CT scans were used in most cases to segment the liver and tumor(s), and to delineate the hepatic and portal venous trees. For CT imaging, iodide contrast medium was used, and the portal-venous phase was chosen for accurate delineation of the liver, tumor(s), and hepatic and portal veins. Scan protocols and resolutions varied between patients because CT scans originated from various referral centers. In one patient, a four-phase contrast-enhanced CT was used for adequate reconstruction of the hepatic arteries which was of specific interest in this case. Additionally, diagnostic contrast-enhanced MR imaging with gadoterate (Dotarem^®^, Guerbet Nederland B.V. Gorinchem, The Netherlands) or gadoxetate (Primovist^®^, Bayer Medical Care B.V. Maastricht, The Netherlands) was used in three cases to delineate tumor tissue, and in one case, a positron emission tomography (PET-)CT scan was used to aid in tumor delineation because contrast-enhanced CT scans were acquired more than four weeks prior to surgery. For all segmentations, the scan with the highest resolution was chosen for tumor delineation. A schematic overview of the segmentation process and implementation of the 3D model in the Robotic Liver Surgery Cockpit is provided in Figure 1.

#### 2.2.2. Clinical Measurements and Preoperative Planning

Segmentations were performed by ODB and AB, and were verified by an experienced radiologist (MCB) and hepatobiliary surgeon (RJS). Tumor sizes in three planes, i.e., longest tumor diameters, were measured by an independent radiologist (RH), blinded to the results of the tumor segmentation and 3D measurements. The longest diameter, measured on either the axial, coronal or sagittal view, was chosen to compare to the major axis measured using the 3D software. Longest tumor diameters on the 3D model were calculated automatically in the ParaView software by selecting the bounding ruler filter to measure the major axis diameter (i.e., the largest 3D diameter). Subsequently, the ‘2D’ measurements were compared to automated measurements of the 2D contours in the axial plane and to the automated measurements in the 3D reconstruction.

#### 2.2.3. Intraoperative Multimodality Imaging Platform—The Robotic Liver Surgery Cockpit

Intuitive’s da Vinci^®^ surgical robot (Intuitive, Sunnyville, CA, USA) contains a platform enabling surgeons to visualize up to three screens (tiles) at the same time using the integrated Tilepro software. In this study, we implemented the interactive virtual reality 3D reconstruction of preoperative CT, MR, and/or PET-CT imaging. By switching on the various tiles, the surgeon was able to simultaneously see the operation field, 3D imaging, fluorescence imaging, and intraoperative ultrasonography (IOUS). All resections were performed following the hospital’s standard of care procedure, using IOUS and near-infrared (NIR) fluorescence with indocyanine green (ICG) as guidance for resection. All patients received 10 mg of ICG approximately 24 h prior to surgery. During the surgical procedure, the surgeon could easily switch views from the visible light operation field to the ‘firefly’ mode (i.e., NIR fluorescence). NIR fluorescence was used for lesion detection, resection planning, and resection margin assessment.

### 2.3. Questionnaire

After each surgical procedure, the surgeon was asked to complete a questionnaire to score various items concerning the applicability and feasibility of the 3D reconstruction in the da Vinci^®^ surgical robot. The questionnaire consisted of the following five statements and two questions: I. The 3D reconstruction helped me to accurately plan the operation when compared to 2D CT or MR imaging. II. Localization of tumors was more accurate using the 3D reconstruction compared to conventional 2D CT images. III. The quality of the 3D reconstruction was high enough for clinical decision-making. IV. I could well assess the proximity of tumors to important surrounding vital structures on the 3D reconstruction. V. The performed surgical treatment was according to preoperative 3D planning. VI. Which (vital) structures were inadequate? VII. In which aspect of clinical decision-making was the 3D reconstruction most beneficial? Statements were scored in the range: totally disagree, disagree, neutral, agree, totally agree, and questions were answered by a predefined multiple choice.

### 2.4. Data Analysis

Validation data of the 3D rendering software was analyzed measuring the interobserver variability using the Dice Similarity Coefficient (DSC), the volume-based Dice Similarity Coefficient (vDSC), and the Degree of Similarity (DoS), where (v)DSC of 1.00 corresponds to a perfect overlap, and where a (v)DSC of 0.00 indicates no overlap. A (v)DSC higher than 0.70 is considered to be a good interobserver agreement.

The correlations of measurements in the radiology workstation, automated measurements in the 3DeliverS software, and automated 3D measurements were calculated using the paired samples t-test. For follow-up data, means, medians, and percentages were calculated.

All statistical analyses were performed using SPSS software Version 25.0 (IBM, New York, NY, USA). Statistical outcomes were significant when the *p*-value was lower than 0.05.

## 3. Results

### 3.1. Software Validation

To ensure the accuracy of our in-house developed segmentation software, we first executed a validation study using the NEMA-2012 phantom. Except for two diameter measurements, all measurements were in range of the actual sizes ± margin of error according to the NEMA-2012 specifications. Taking the in-plane resolution (1.17 mm × 1.17 mm) with a slice thickness of 0.67 mm into account, all measurements were well in range of the real-life dimensions of the NEMA-2012. Furthermore, an excellent interobserver agreement was achieved. The DoS (within two pixel range) showed perfect resemblance of the segmentations by the independent observers. The 3DeliverS software showed accurate measurements of both volume and largest axial diameter. Both DSC and vDSC showed high interobserver agreement (≥0.87).

Only the largest axial diameter of sphere 6 from observer 1 (10.6 mm) and the largest axial of sphere 1 from observer 2 (38.2 mm) were out of range of the NEMA-2012 specifications (Table 1). In addition, the largest axial contour was slightly overestimated by the observers, probably due to the thickness of the sphere’s wall. The thickness is smaller than the in-plane resolution, which makes it difficult to determine the true inside edges of the filled spheres. The interobserver variability was larger in smaller spheres. This might be caused by the voxel size relative to the size of the spheres. The upper and lower edges of the smaller spheres were relatively difficult to visualize compared to larger spheres.

### 3.2. Implementation

#### 3.2.1. Imaging Modalities and 3D Modeling

For all 15 patients, contrast-enhanced CT scans were available for liver and tumor delineation. Additionally, diagnostic contrast-enhanced MRI scans were used in three patients and PET-CT scan in one patient to delineate tumors.

#### 3.2.2. Intraoperative Implementation of the Robotic Liver Surgery Cockpit

**Figure 2:**
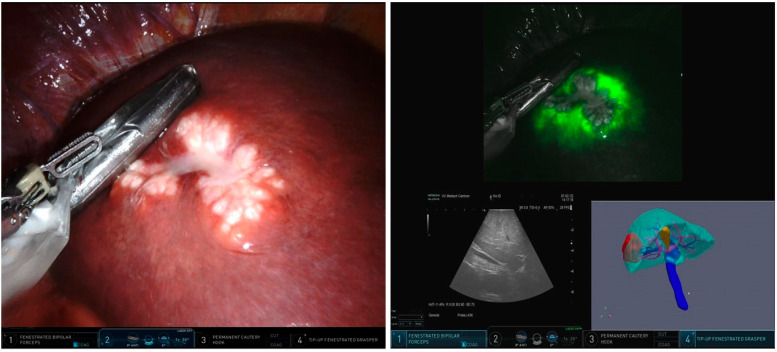
**Surgeon’s perspective using the multimodality Robotic Liver Surgical Cockpit.** (**Left**) image showing a white light image of liver and tumor in segment 6/7; (**Right**) image showing the near-infrared fluorescence overlay image of the tumor site in segment 6/7 in the upper panel; intraoperative ultrasonography of the tumor in the lower left panel and the Virtual Reality 3D model of the liver, tumor, and vital structures in the lower right panel.

After several dry-lab tests, the 3D model was installed in the clinical surgical cockpit available in the skills laboratory in our institution. At first, the 3D model was opened in ParaView and connected to the Tilepro via a USB-c to DVI cable. A laptop was used to navigate through the 3D reconstruction in the first 10 patients, for the last 5 patients, a tablet mounted to the da Vinci surgical robot was used for more user-friendly and intuitive navigation. The surgeon’s perspective of the multimodality imaging platform is displayed in Figure 2.

#### 3.2.3. Surgical Outcomes and Clinical Measurements

A total of 15 patients were included in the study. Patients were scheduled for a variety of anatomic or non-anatomic robot-assisted liver resection. In these procedures, there was no conversion to open surgery, and no intraoperative incidents occurred according to the Oslo Classification of Intraoperative Unfavorable Incidents [19]. In one patient, the tumor was judged to be non-resectable. Of the 15 patients, 13 patients had colorectal liver metastases, 1 patient had intrahepatic cholangiocarcinoma, and 1 patient had focal nodular hyperplasia. Of the 22 resected lesions, 21 were confirmed malignant after histopathological assessment, and all malignant lesions were CRLM. Four (19%) of 21 malignant lesions had positive tumor margins (R1), whereas 17 (81%) were radical resections (R0). Three out of four R1 resections were intentional vascular R1 resections and showed positive ICG-fluorescence signal in the resection plane. Patient characteristics, operation time, estimated blood loss, length of hospital stay, and resection margin status are summarized in Table 2.

Longest tumor diameters on CT and MR imaging did not differ significantly (35.83 mm vs. 35.13 mm, respectively; *p* = 0.415) compared to the automated measurements of the contours in the axial plane from in the 3DeliverS software. Furthermore, longest tumor diameters were also comparable to automated major tumor axis measurements in the ParaView software (35.83 mm vs. 36.65 mm, respectively; *p* = 0.70) (Table 3).

#### 3.2.4. Questionnaire

**Figure 3:**
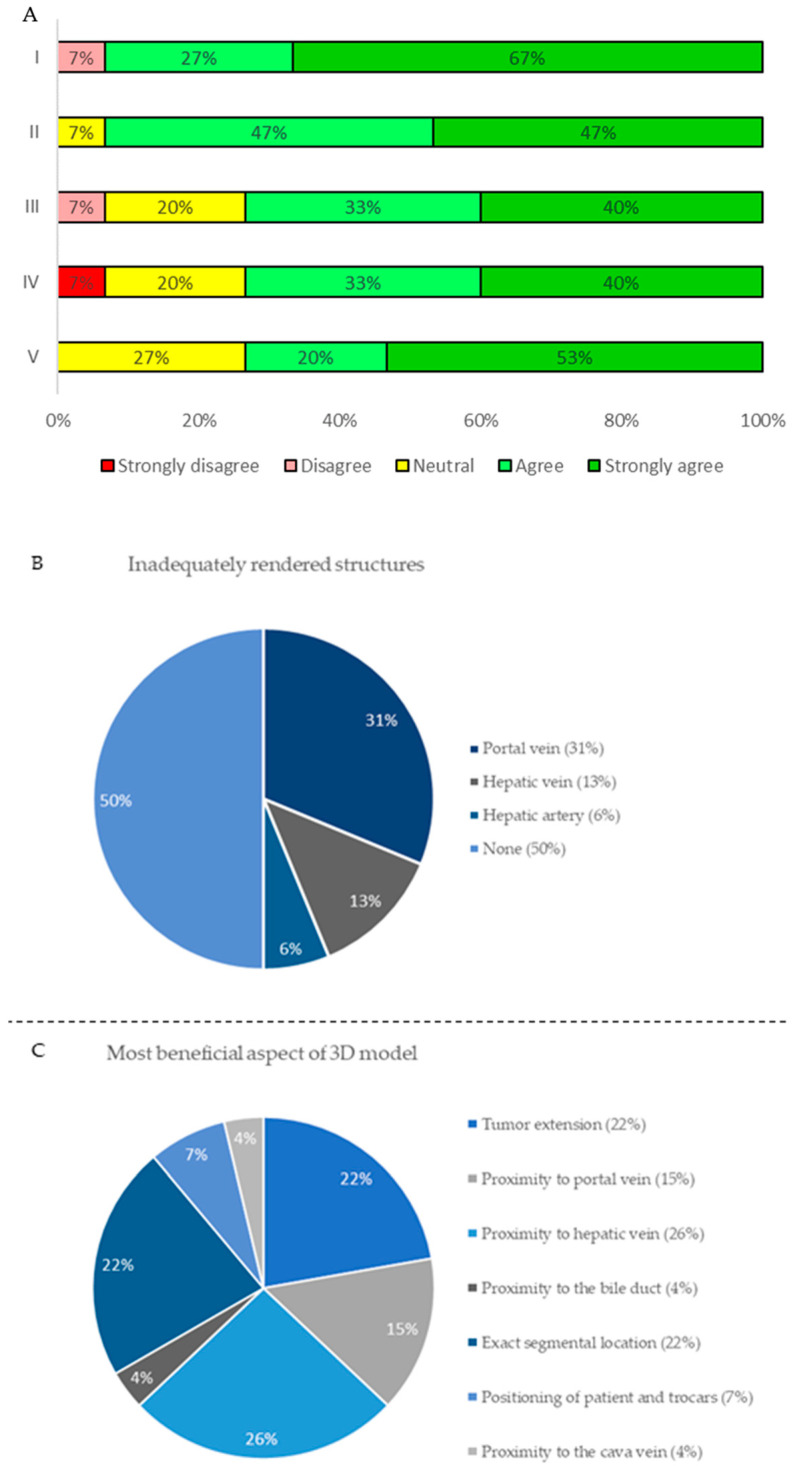
**Chart results of the Questionnaire.** (**A**) Stacked bar chart of Likert scale Questionnaire results displaying overall satisfaction with the 3D reconstructions used of the 15 patients in this feasibility study. (I) the 3D reconstruction helped me to accurately plan the operation when compared to 2D CT or MR imaging; (II) localization of tumors was more accurate using the 3D reconstruction compared to conventional 2D CT images; (III) the quality of the 3D reconstruction was high enough for clinical decision-making; (IV) I could well assess the proximity of tumors to important surrounding vital structures on the 3D reconstruction; (V) the performed surgical treatment was according to preoperative 3D planning. (**B**,**C**) Pie charts of the two multiple choice questions of the questionnaire displaying the most frequent inadequacies (**B**) and the most beneficial aspects (**C**) of the 3D reconstructions.

The questionnaire was completed postoperatively for all 15 patients by the surgeon performing the resection. Overall, the surgeon was highly satisfied with the 3D models in 14 out of 15 cases (93%). An overview of the Likert scale-based questionnaire is displayed in Figure 3A. The portal vein and its branches, and the hepatic vein were missing in 5 (33%) cases and 3 (20%) cases, respectively, and were therefore the most frequent limiting factor for clinical decision-making (Figure 3B). The proximity of the tumor to the hepatic vein, tumor extension, and exact segmental location were the most frequent beneficial aspects of the 3D models (Figure 3C).

## 4. Discussion

Surgeons benefit from additional tools for preoperative planning and intraoperative guidance for robotic liver surgery. In this study, we successfully introduced the Robotic Liver Surgery Cockpit, a multimodality imaging platform including virtual reality 3D rendered models created with the in-house developed 3DeliverS software.

The software validation experiment showed that segmentations and measurements with 3DeliverS are in accordance with real-life dimensions as all measurements, of both volume and largest axial contours, were in range of the NEMA-2012 dimensions ± uncertainty due to the CT resolution. In addition, the experiment showed that for clearly visible spheres, the interobserver agreement is excellent with a DSC ≥ 0.87, a vDSC ≥ 0.89, and a DoS ≥ 0.99.

Subsequently, the segmented CT, MR, and PET-CT imaging of patients with both malignant and benign liver lesions were successfully implemented as virtual reality 3D models in the surgical console. Connecting various devices to Intuitive’s da Vinci surgical console was very straightforward, thus making the techniques used in this feasibility study accessible and easy to use for each robotic surgeon.

The segmentation of the liver and tumors was accurate when comparing longest tumor diameters measured on CT and MR imaging, in the 3DeliverS segmentation software, as well as in the 3D application. Although not significant, the automated measurements in 3D were higher compared to those in a two-dimensional plane. This may be attributed to the fact that tumors may have erratic growth patterns which are hard to measure in one two-dimensional slice. The 3D measurements can also take non-orthogonal image planes into account to obtain the largest diameter.

Moreover, results of the questionnaire clearly showed that the virtual reality 3D model is user-friendly and aids the surgeon during various phases of the operation, similar to previous reports of open liver surgery [10,11,12,13]. Preoperative planning with virtual reality 3D models creates improved visualization of the exact location of tumors relative to vital hepatic anatomy resulting in a change of positioning of the patients and robotic trocars. Furthermore, the virtual reality 3D models are supportive in planning of the parenchymal transaction based on extension of the tumors in liver parenchyma. Most commonly, the segmentation of the portal vein branches were insufficient, making the virtual reality 3D model less reliable in some cases.

In this study, all surgical procedures were conducted without intraoperative incidents and intraoperative parameters were comparable to previous reports [2,3,4]. We reported 19% R1 resections, however, three out of four R1 resections were intentional vascular R1 resections, which have comparable reported outcomes to parenchymal R0 resections [20]. 

Using this multimodal imaging approach consisting of a virtual reality 3D model, IOUS and NIR fluorescence with ICG surgeons simultaneously have all preoperative and intraoperative information at their disposal, potentially leading to shorter operation time, a decrease in intraoperative blood loss, and improved patient outcomes [21,22]. Larger cohorts and randomized trials in robotic partial nephrectomy have confirmed these findings [17,18] but need to be investigated for robot-assisted liver surgery. Although both the subjective and objective results from this feasibility study seem promising, larger case-matched or randomized trials, with clear surgical endpoints (e.g., blood loss, length of hospital stay) or oncologic endpoints (e.g., resection margin status, recurrence-free survival and overall survival) are required to examine whether preoperative planning and intraoperative guidance using virtual reality 3D models in robot-assisted liver surgery leads to change in surgical management and subsequently improves patient’s outcome.

### Limitations

Several limitations should be addressed. First, due to the small sample size of this feasibility study, and the absence of a control group, no statistical comparison could be performed on intraoperative and postoperative outcomes. Second, CT and MRI scans from various hospitals were used, therefore no rigid scan protocol was used leading to high variability in quality and resolutions. For further development of the automatic segmentation methods for the liver and intrahepatic structures a standardized CT and MRI contrast and scan protocol is required. In this study we implemented an interactive virtual reality 3D model. Third, although rendering was adequate and feasible in the entire study cohort, this application lacks real-time visualization. As a next step, focus should also be put on the feasibility and applicability of augmented reality with the motion compensation for real-time planning and guidance using 3D overlays. These next steps may be challenging due to continuous movement of the liver and surrounding structures as a result of patient’s respiration and the manipulation of the organ by the surgeon. Although several case reports already described this technique, numerous developmental steps need to be made before augmented reality can be implemented as a safe and reliable additional tool during robotic liver surgery [14,16].

## 5. Conclusions

Segmentation and 3D modeling with in-house development of preoperative CT and MRI scans is feasible, and the da Vinci surgical robot is a highly suitable platform for the implementation of multimodal imaging of virtual reality 3D models, intraoperative ultrasonography, and ICG-fluorescence. Virtual reality 3D models may improve preoperative planning, patient positioning, robotic trocar positioning, and intraoperative guidance. Further developments and research should focus on improved automatic segmentation of various structures, the clinical impact in terms of patients’ outcomes, and the role of real-time augmented reality overlay imaging tools.

## Figures and Tables

**Table 1:** Measurements and interobserver variability of the segmentation results versus real-life dimensions of the NEMA-2012 PET phantom.

	Sphere 1	Sphere 2	Sphere 3	Sphere 4	Sphere 5	Sphere 6
**NEMA-2012**						
*Volume* (mm^3^) *Largest axial diameter* (mm)	26,522 ± 2151 37.0 ± 1	11,494 ± 1232 28.0 ± 1	5575 ± 761 22.0 ± 1	2572 ± 227 17.0 ± 0.5	1150 ± 133 13.0 ± 0.5	524 ± 79 10.0 ± 0.5
**Observer 1**						
*Volume* (mm^3^) *Largest axial diameter* (mm)	25,342 37.4	11,570 28.1	5230 22.3	2319 17.5	1215 13.4	522 10.6
**Observer 2**						
*Volume* (mm^3^) *Largest axial diameter* (mm)	26,921 38.2	11,463 28.5	5874 22.3	2624 17.7	1188 13.5	521 10.4
**DSC** **vDSC** **DoS**	0.96 0.96 1.00	0.95 0.96 1.00	0.92 0.94 1.00	0.88 0.91 0.99	0.90 0.93 1.00	0.87 0.89 1.00

(v)DSC = (volume-based) Dice Similarity Coefficient; DoS = Degree of Similarity.

**Table 2:** Patient characteristics and clinical data.

Patient Characteristics	
Age at surgery, median, (IQR)	64
Sex, No. (%) Male Female	11 (73) 4 (27)
Histological diagnosis, No. (%) CRLM CCA FNH	13 (87) 1 (6.5) 1 (6.5)
No. of target lesions (mean)	23 (1.5)
No. malignant lesions resected (mean) R0 (%) R1 (%) Expected R1 based on ICG (%)	21 (1.6) 17 (81) 4 (19) 3 (75)
Type of resection, No. (%) Anatomic Non-anatomic N.A.	5 (33) 9 (60) 1 (7)
Operation time (min), median (IQR)	192 (135–263)
Estimated blood loss (mL), median (IQR)	200 (10–350)
Length of stay (days), mean (SD)	3 (3.035)

**Table 3:** Longest tumor diameter measurements in conventional radiology working station, as segmented in the 3DliverS software, and automated measurements in 3D model.

	Longest Tumor Diameter (mm)	Mean Difference (mm)	*p*-Value (95% CI)	Correlation
**On CT** **3DeliverS**	35.83 35.13	0.696	0.265 (−0.565–1.956)	0.989 (*p* < 0.01)
**On CT** **3D measurement**	35.83 36.65	0.826	0.416 (−2.894–1.242)	0.973 (*p* < 0.01)

## Data Availability

The data presented in this study are available on request from the corresponding author.

## References

[1] Lim C., Salloum C., Tudisco A., Ricci C., Osseis M., Napoli N., Lahat E., Boggi U., Azoulay D. (2019). Short- and Long-term Outcomes after Robotic and Laparoscopic Liver Resection for Malignancies: A Propensity Score-Matched Study. World J. Surg..

[2] Beard R.E., Khan S., Troisi R.I., Montalti R., Vanlander A., Fong Y., Kingham T.P., Boerner T., Berber E., Kahramangil B. (2020). Long-Term and Oncologic Outcomes of Robotic Versus Laparoscopic Liver Resection for Metastatic Colorectal Cancer: A Multicenter, Propensity Score Matching Analysis. World J. Surg..

[3] Zhang L., Yuan Q., Xu Y., Wang W. (2020). Comparative clinical outcomes of robot-assisted liver resection versus laparoscopic liver resection: A meta-analysis. PLoS ONE.

[4] Fretland Å.A., Dagenborg V.J., Bjørnelv G.M.W., Kazaryan A.M., Kristiansen R., Fagerland M.W., Hausken J., Tønnessen T.I., Abildgaard A., Barkhatov L. (2018). Laparoscopic Versus Open Resection for Colorectal Liver Metastases: The OSLO-COMET Randomized Controlled Trial. Ann. Surg..

[5] Spiegelberg J., Iken T., Diener M.K., Fichtner-Feigl S. (2022). Robot-assisted Surgery for Primary Hepatobiliary Tumors-Possibilities and Limitations. Cancers.

[6] Renzulli M., Clemente A., Ierardi A.M., Pettinari I., Tovoli F., Brocchi S., Peta G., Cappabianca S., Carrafiello G., Golfieri R. (2020). Imaging of Colorectal Liver Metastases: New Developments and Pending Issues. Cancers.

[7] Beermann J., Tetzlaff R., Bruckner T., Schöebinger M., Müller-Stich B.P., Gutt C.N., Meinzer H.P., Kadmon M., Fischer L. (2010). Three-dimensional visualisation improves understanding of surgical liver anatomy. Med. Educ..

[8] Michels N.A. (1966). Newer anatomy of the liver and its variant blood supply and collateral circulation. Am. J. Surg..

[9] Sureka B., Patidar Y., Bansal K., Rajesh S., Agrawal N., Arora A. (2015). Portal vein variations in 1000 patients: Surgical and radiological importance. Br. J. Radiol..

[10] Lamata P., Lamata F., Sojar V., Makowski P., Massoptier L., Casciaro S., Ali W., Stüdeli T., Declerck J., Elle O.J. (2010). Use of the Resection Map system as guidance during hepatectomy. Surg. Endosc..

[11] Mise Y., Hasegawa K., Satou S., Shindoh J., Miki K., Akamatsu N., Arita J., Kaneko J., Sakamoto Y., Kokudo N. (2018). How Has Virtual Hepatectomy Changed the Practice of Liver Surgery?: Experience of 1194 Virtual Hepatectomy Before Liver Resection and Living Donor Liver Transplantation. Ann. Surg..

[12] Fang C.H., Tao H.S., Yang J., Fang Z.S., Cai W., Liu J., Fan Y.F. (2015). Impact of three-dimensional reconstruction technique in the operation planning of centrally located hepatocellular carcinoma. J. Am. Coll. Surg..

[13] Zeng N., Tao H., Fang C., Fan Y., Xiang N., Yang J., Zhu W., Liu J., Guan T., Fang C. (2016). Individualized preoperative planning using three-dimensional modeling for Bismuth and Corlette type III hilar cholangiocarcinoma. World J. Surg. Oncol..

[14] Buchs N.C., Volonte F., Pugin F., Toso C., Fusaglia M., Gavaghan K., Majno P.E., Peterhans M., Weber S., Morel P. (2013). Augmented environments for the targeting of hepatic lesions during image-guided robotic liver surgery. J. Surg. Res..

[15] Volonté F., Pugin F., Buchs N.C., Spaltenstein J., Hagen M., Ratib O., Morel P. (2013). Console-integrated stereoscopic OsiriX 3D volume-rendered images for da Vinci colorectal robotic surgery. Surg. Innov..

[16] Pessaux P., Diana M., Soler L., Piardi T., Mutter D., Marescaux J. (2015). Towards cybernetic surgery: Robotic and augmented reality-assisted liver segmentectomy. Langenbecks Arch. Surg..

[17] Shirk J.D., Kwan L., Saigal C. (2019). The Use of 3-Dimensional, Virtual Reality Models for Surgical Planning of Robotic Partial Nephrectomy. Urology.

[18] Shirk J.D., Thiel D.D., Wallen E.M., Linehan J.M., White W.M., Badani K.K., Porter J.R. (2019). Effect of 3-Dimensional Virtual Reality Models for Surgical Planning of Robot-assisted Partial Nephrectomy on Surgical Outcomes: A Randomized Clinical Trial. JAMA Netw. Open.

[19] Kazaryan A.M., Røsok B.I., Edwin B. (2013). Morbidity assessment in surgery: Refinement proposal based on a concept of perioperative adverse events. ISRN Surg..

[20] Viganò L., Procopio F., Cimino M.M., Donadon M., Gatti A., Costa G., Del Fabbro D., Torzilli G. (2016). Is Tumor Detachment from Vascular Structures Equivalent to R0 Resection in Surgery for Colorectal Liver Metastases? An Observational Cohort. Ann. Surg. Oncol..

[21] Achterberg F.B., Sibinga Mulder B.G., Meijer R.P.J., Bonsing B.A., Hartgrink H.H., Mieog J.S.D., Zlitni A., Park S.M., Farina Sarasqueta A., Vahrmeijer A.L. (2020). Real-time surgical margin assessment using ICG-fluorescence during laparoscopic and robot-assisted resections of colorectal liver metastases. Ann. Transl. Med..

[22] Handgraaf H.J.M., Boogerd L.S.F., Hoppener D.J., Peloso A., Sibinga Mulder B.G., Hoogstins C.E.S., Hartgrink H.H., van de Velde C.J.H., Mieog J.S.D., Swijnenburg R.J. (2017). Long-term follow-up after near-infrared fluorescence-guided resection of colorectal liver metastases: A retrospective multicenter analysis. Eur. J. Surg. Oncol. J. Eur. Soc. Surg. Oncol. Br. Assoc. Surg. Oncol..

